# Cellular transport of anti-inflammatory pro-drugs originated from a herbal formulation of *Zingiber cassumunar *and *Nigella sativa*

**DOI:** 10.1186/1749-8546-4-19

**Published:** 2009-09-25

**Authors:** Prasan Tangyuenyongwatana, Jariya Kowapradit, Praneet Opanasopit, Wandee Gritsanapan

**Affiliations:** 1 Department of Pharmacognosy, Faculty of Pharmacy, Mahidol University, Bangkok 10400, Thailand; 2 Department of Pharmaceutical Technology, Faculty of Pharmacy, Silpakorn University, Nakhon Pathom 73000, Thailand

## Abstract

**Background:**

The rhizome of *Zingiber cassumunar *and the seed of *Nigella sativa *are two ingredients in Thai traditional medicine to relieve dysmenorrhea and adjust the menstrual cycle. Mixture of these two herbs produces three esters, namely (*E*)-4-(3,4-dimethoxyphenyl)but-3-en-1-yl linoleate (1), (*E*)-4-(3,4-dimethoxyphenyl)but-3-en-1-yl oleate (2) and (*E*)-4-(3,4-dimethoxyphenyl)but-3-en-1-yl palmitate (3). The aim of this study is to examine *in vitro *absorption of these esters and evaluate their transport across the membrane.

**Methods:**

*In vitro *transport of these three esters was observed in Caco-2 cell monolayers. The ester compounds 1, 2 and 3 at a concentration of 10 μM were hydrolyzed by porcine liver esterase.

**Results:**

All esters transported across the Caco-2 cell without enzymatic hydrolysis. The apparent permeability coefficients *P*_app _of compound 1 at 53 μM and 106 μM were 13.94 (0.60) × 10^-6 ^and 14.33 (0.17) × 10^-6^cm/s respectively, while those of compound 2 were 9.45 (0.29) × 10^-6 ^and 10.08 (0.32) × 10^-6^cm/s, respectively. *P*_app _values of compound 3 were 7.48 (0.31) × 10^-6^cm/s at 53 μM and 8.60 (0.55) × 10^-6^cm/s at 106 μM. *P*_app _values of the parent compound (compound D), i.e. (*E*)-4-(3,4-dimethoxyphenyl)but-3-en-1-ol were 8.53 (0.83) × 10^-6^cm/s at 53 μM and 16.38 (0.61) × 10^-6^cm/s at 106 μM. The ester hydrolysis of compounds 1, 2 and 3 by porcine liver esterase was monitored by HPLC and the hydrolysis reactions were completed within 10 minutes.

**Conclusion:**

Using the Caco-2 cell monolayer model, the present study finds that compounds (*E*)-4-(3,4-dimethoxyphenyl)but-3-en-1-yl linoleate (1), (*E*)-4-(3,4-dimethoxyphenyl)but-3-en-1-yl oleate (2) and (*E*)-4-(3,4-dimethoxyphenyl)but-3-en-1-yl palmitate (3) originated from Prasaplai preparation (a Thai herbal formula) may be transported through a facilitated mechanism and serve as pro-drugs to increase the compound D level in the blood.

## Background

*Zingiber cassumunar *(*Z. cassumunar*, cassumunar ginger) and *Nigella sativa *(*N. sativa*, black cumin) are widely used as single herbs or as components of herbal formulae in Asian traditional medicines. One of the compounds isolated from *Z. cassumunar*, (*E*)-4-(3,4-dimethoxyphenyl)but-3-en-1-ol is named compound D [1,2]. In a carrageenan-induced rat paw edema model, compound D from a hexane extract of *Z. cassumunar *showed a potent inhibitory effect on edema formation [2]. Compound D also demonstrated a dose dependent uterine relaxant effect in uterus of non-pregnant rat [3].

**Figure 1:**
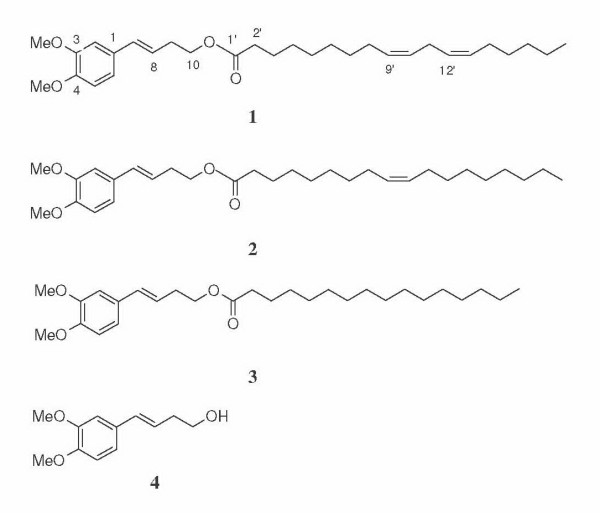
**Chemical structures of compounds 1, 2, 3 and D 1: compound 1; 2: compound 2; 3: compound 3; 4: compound D**.

Three artificial fatty acid esters were found in the mixture of dry powders of *Z. cassumunar *rhizome and *N. sativa *seeds [4,5] which are main constituents of the Prasaplai preparation, a traditional Thai herbal formula to treat dysmenorrheal and adjusting the menstrual cycle [6]. The three artificial fatty acid esters were identified as (*E)*-4-(3,4-dimethoxy-phenyl)but-3-en-1-yl linoleate (1), (*E*)-4-(3,4-dimethoxy-phenyl)but-3-en-1-yl oleate (2) and (*E*)-4-(3,4-dimethoxy-phenyl)but-3-en-1-yl palmitate (3) (Figure 1). Reaction between compound D in *Z. cassumunar *and linoleic, oleic and palmetic acids from *N. sativa *generates these three artificial compounds which are active against *Mycobacterium tuberculosis *H_37_Ra. Minimal inhibitory concentration of compounds 1 and 3 is 200 μg/ml and that of compound 2 is 100 μg/ml. When tested for anti-herpes simplex virus (HSV-1) activities in human vero cell line, compound 2 was active at IC_50 _of 42.6 μg/ml without cytotoxicity whereas compound 3 was cytotoxic at IC_50 _of 38 μg/ml [7].

We are interested in the role of these fatty acid esters in the Prasaplai preparation. One hypothesis is that the fatty acid esters may act as pro-drugs in order to increase the absorption [8] of the parent compound, i.e. compound D. The present study aims to investigate the absorption of these fatty acid esters in an *in vitro *model and predict their transport across the human intestinal membrane.

As a cell monolayer model that imitates *in vivo *intestinal epithelium in human Caco-2 cell line, a human colon adrenocarcinoma grows rapidly into confluent monolayer that exhibits several characteristics of differentiated epithelial cells. Permeation characteristic of compounds especially drugs across Caco-2 cell monolayer correlates with their human intestinal mucosa permeation characteristics [9]. We used this cell culture model to assess the intestinal permeability of tested compounds.

## Methods

### Materials

Compounds 1, 2, 3 and D were synthesized and purified (over 95%) in our laboratory [5]. The Caco-2 cell line was obtained from the American Type Culture Collection (ATCC HTB-37). Dulbecco's modified Eagle's medium (DMEM), trypsin-EDTA, L-glutamine, non-essential amino acid, penicillin-streptomycin antibiotics and fetal bovine serum (FBS) were obtained from GIBCO-Invitrogen (USA). Transwell (6-well plates) cell culture chambers inserted with 3.0 μm pore size were purchased from Corning Life Sciences (USA). Esterase enzyme was obtained from Sigma (USA). All other chemicals were of cell culture and molecular biology grade from Sigma (USA).

### Analytical methods

We used a high performance liquid chromatography (HPLC) system consisting of a Knauer pump K-1001 and a Knauer Photometer K-2600 detector (Knauer, Germany) with detection at 254 nm. The separation was performed on a Kromasil 5 μm 100AC_18_, 250 × 4 mm column (Phenomenex, USA). Flow rate was 0.8 ml per minute and the solvent system was a gradient elution of 1% acetic acid in water and acetonitrile (CH_3_CN) at 85:15, 70:30, 55:45, 50:50, 30:70, 15:85, 0:100 and 0:100 at 0, 8, 25, 30, 55, 65, 80 and 110 minutes, respectively. Compounds 1, 2 and 3 were separated by non-polar stationary phase (octadecylsilane, ODS) eluted with 100% CH_3_CN at the last stage of the gradient elution.

As the fatty acid esters were not stable in transported medium, the UV spectroscopy was used to monitor the amounts of compounds 1, 2, 3 and D. Spectrophotometry analysis was performed on a Helios alpha UV-Vis spectrophotometer (Thermo Scientific, USA). The maximum wavelength (λ_max_) was obtained at 228 nm. Standards of each compound were freshly prepared at the concentration range of 0.62-3.73 μg/mL. Validations were performed by five replicates of intra-day and three replicates of inter-day. The linear correlation coefficients (*r*) between the UV-absorption and the concentrations of all compounds were in the range of 0.9993-0.9996 (compound 1: Y = 109.67X+0.031, *r *= 0.9994; compound 2: Y = 109.44X+0.028, *r *= 0.9995; compound 3: Y = 118.58X+0.049, *r *= 0.9996; compound D: Y = 169.72X+0.058, *r *= 0.9993).

### Cell culture

Caco-2 cells were maintained in a DMEM at pH7.4, supplemented with 10% fetal bovine serum, 2 mM L-glutamine, 1% non-essential amino acid solution and 0.1% penicillin-streptomycin solution in a humidified atmosphere (5% CO_2_, 95% air, at 37°C). Cells were grown until 60-70% confluence. Cells from passages 20-40 were used for all experiments. Cells were seeded on tissue culture polycarbonate membrane filters (pore size: 3.0 μm) in 6-well Transwell plates (Corning, USA) at a seeding density of 2 × 10^4^cells/cm^2^. Culture medium was added to both the donor and the acceptor compartments. Medium was changed every two days. Cells were left to differentiate for 15-21 days after seeding with monitoring of trans-epithelial electrical resistance (TEER) values with a Millicell electrical resistance system (Millipore, USA) and the value should be higher than 600Ω.cm^2^.

### Chemical hydrolysis study of the compounds in transport medium

Solution of compound 1, 2 and 3 at 53 μM and 106 μM were prepared in Hank's balanced salt solution (HBSS) at pH7.4. All solutions were kept at 4°C for 96 hours and each solution was then analyzed by HPLC at 12, 24, 48, 72 and 96 hours to determine the hydrolysis product.

### Transport studies

Transport experiment across the Caco-2 cell monolayers at pH7.4 was performed. Caco-2 cell monolayers in Transwell (6-well) plates were used for transport studies when they were differentiated and the monolayer was intact, as checked by measuring TEER. Prior to the experiment, the cells were washed twice with phosphate buffered saline (PBS) and pre-equilibrated for one hour with HBSS buffered with 30 mM n-(2-hydroxyethyl) piperazine-n-(2-ethanosulfonic acid) (HEPES) at pH7.4. After medium was removed, the cells were treated with sample solutions (concentrations of 53 μM and 106 μM in HBSS at pH7.4) in an apical compartment. Samples (1 mL) were taken under sink conditions at 0, 5, 20, 40, 60, 80 and 100 minutes from the basolateral side and replaced with an equal volume of fresh HBSS solution. The amount of the compounds from the basolateral side was determined on a UV-spectrophotometer (Thermo Scientific, USA) at 228 nm. Results were expressed as cumulative transport as a function of time. Apparent permeability coefficient was calculated according to the following equation:



where *P*_app _is the apparent permeability coefficient (cm/s), d*Q*/d*t *(μg/s) is the rate of appearance of sample on the basolateral side, *A *is the surface area of the monolayer (cm^2^) and *C*_0 _(μg/mL) is the initial drug concentration in the donor compartment. All rate constants were obtained from the permeation profiles of each compound. Statistical significance was evaluated with one-way analysis of variance (one-way ANOVA). A value of *P *< 0.05 was considered statistically significant.

### Enzyme hydrolysis study of the compounds

Compound 1 (2 mL, 10 μM solution) was pre-incubated at 37°C and 200 μL of esterase enzyme (porcrine liver, 750 units) was added. Samples (200 μL each) were taken at 5, 10, 20, 30 and 60 minutes and added to 200 μL of methanol. Mixtures were vortexed to stop enzymatic activity. Samples were then centrifuged for five minutes at 14,000 × g (Lab Essentials, USA). Supernatant was injected to the HPLC system for the determination of ester pro-drugs and compound D. This procedure was repeated for compounds 2 and 3.

## Results

Compounds 1, 2 and 3 were prepared in transport medium at concentrations of 53 μM and 106 μM and stored at 4°C for 96 hours. The samples were collected every 12 hours and analyzed on the HPLC system. Compound D was detected by the HPLC after 24 hours. As the fatty acid esters were not stable long enough in the transported medium, the HPLC analysis must be carried out in a short period of time. A UV absorption method was used to measure the amount of transported compounds in this experiment. The UV analysis was designed and validated in the range of 0.62-3.73 μg/mL and the linear correlations (*r*) of all compounds were shown in the range of 0.9993-0.9996. All samples were analyzed and finished within several hours.

**Figure 2:**
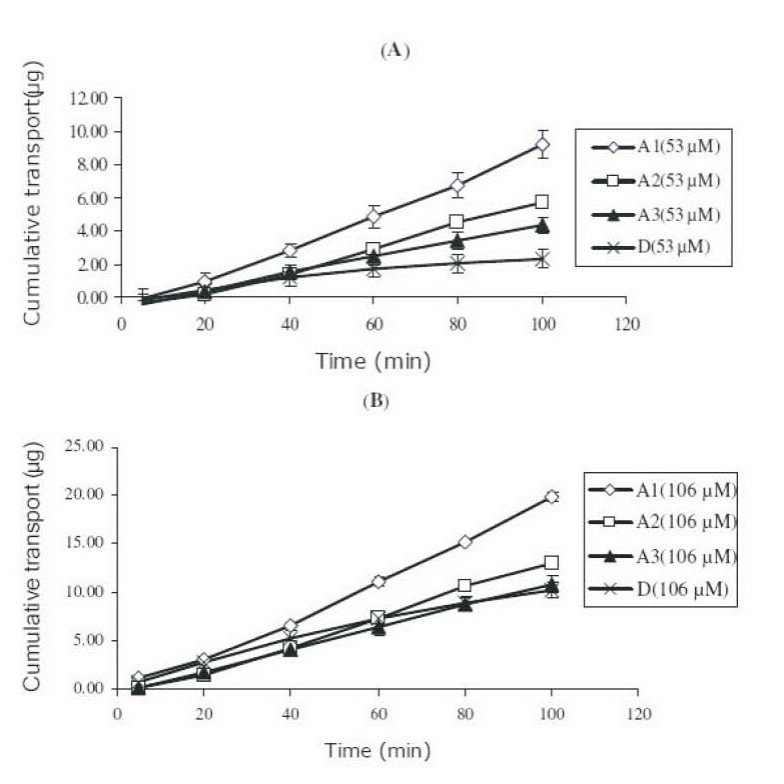
**Cumulative amounts of the fatty acid esters and compound D**. (A) At 53 μM in the apical to basolateral direction, compound 1 (A1) showed highest cumulative transport over other two fatty acid ester (A2 and A3) and compound D. (B) At 106 μM in the apical to basolateral direction, compound 1 (A1) showed higher cumulative transport over other two fatty acid ester (A2 and A3) while compound D had cumulative transport close to compound 3 (A3). A1: compound 1; A2: compound 2; A3: compound 3; D: compound D.

Rate of transport of each compound was estimated from the slope of the linear portion of a plot of cumulative amount. The apparent permeability coefficients (*P*_app_) for these compounds were calculated from the experimental data which were determined from the apical to basolateral side (Table 1, Figure 2).

**Table 1:** Apparent permeability coefficient (*P*_app_) of compounds 1, 2, 3 and D (n = 3)

**Compound**	*P_app_**at 53 μM** **(10**^-6^**cm/s)**	*P_app_**at 106 μM** **(10**^-6^**cm/s)**	***P*** **Value**
Compound 1	13.94 (0.60)	14.33 (0.17)	0.364
Compound 2	9.45 (0.29)	10.08 (0.32)	0.146
Compound 3	7.48 (0.31)**	8.60 (0.55)**	0.014
Compound D	8.53 (0.83)**	16.38 (0.61)**	0.001

*expressed as mean (SD), n = 3

**P < 0.05, significantly different between these two concentrations.

The hydrolysis of the fatty acid esters was confirmed by an *in vitro *assay with esterase enzyme from porcine liver. After the esters were mixed with esterase enzyme and incubated at 37°C, the hydrolysis reaction was completed within 10 minutes and compound D was detected.

The apparent permeability coefficient (*P*_app_) of compounds 1 and 2 at 53 μM showed no significant difference compared with those at concentration of 106 μM (compound 1: *P *= 0.364; compound 2: *P *= 0.146). *P*_app _of compound D at 106 μM was significantly different from that at 53 μM (*P *= 0.001). Compound D had *P*_app _values close to those of compounds 2 and 3 at 53 μM, while *P*_app _values of compounds D, 1, 2 and 3 differed significantly (*P *= 0.001) at 106 μM.

The lipophilicity of these compounds can be estimated with software ACD/labs version 11.03 (Advanced Chemistry Development, USA). The log P of compounds 1, 2, and 3 were 10.13 (0.42), 10.65 (0.40) and 10.11 (0.39) respectively.

## Discussion

The results suggest that the absorption mechanism of compounds 1 and 2 was not dependent on the concentrations of the fatty acid esters. Facilitated transport may be the mechanism. Compound D seemed to follow a concentration dependent passive diffusion mechanism.

*P*_app _values ranged from 7.47 × 10^-6 ^to 16.38 × 10^-6 ^cm/s in an apical to basolateral direction. According to a previous study [10], *P*_app _value in Caco-2 cells higher than 1 × 10^-6^cm/s is associated with efficient intestinal absorption in human. At 53 μM, compound 1 showed the highest transport across the Caco-2 cell monolayers among all compounds. Compound 2 demonstrated higher permeability than that of compound D (*P *= 0.146). At 106 μM, compound 1 showed higher transport across Caco-2 cell than that of compounds 2 and 3 but lower transport than that of compound D.

**Figure 3:**
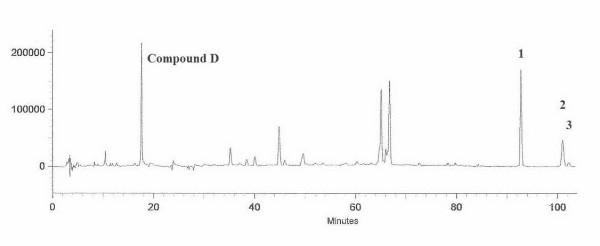
**HPLC chromatograms of the mixture of *Z. cassumunar *and *N. sativa***. 1: compound 1; 2: compound 2; 3: compound 3.

These lipophilicity values do not correlate well to the transport results. More valid estimation of lipophilicity of the compounds may come from analysis of retention time of a reverse phase HPLC chromatogram [11,12] (Figure 3). The retention times (t_r_) of compounds 1, 2 and 3 were 92.4, 101.0 and 102.0 minutes, respectively. Polarity of compound 1 was higher than that of compounds 2 and 3.

The hydrolysis of compound 1, 2 and 3 did not occur during the transport across Caco-2 cell monolayers. This may be due to the unusual long chain fatty acid structure of these compounds and other factors such as the duration of the time contact with the enzyme during transport. However the hydrolysis of the ester linkage in these compounds may occur in the blood circulation or in the liver because these compounds can be hydrolyzed by porcine liver esterase within in ten minutes.

## Conclusion

Using the Caco-2 cell monolayer model, the present study finds that compounds (*E)*-4-(3,4-dimethoxy-phenyl)but-3-en-1-yl linoleate (1), (*E*)-4-(3,4-dimethoxy-phenyl)but-3-en-1-yl oleate (2) and (*E*)-4-(3,4-dimethoxy-phenyl)but-3-en-1-yl palmitate (3) originated from the Prasaplai preparation (a Thai herbal formula) may be transported through a facilitated mechanism and serve as pro-drugs to increase the compound D level in the blood.

## Abbreviations

HSV-1: herpes simplex virus-1; DMEM: Dulbecco's modified Eagle's medium; FBS: fetal bovine serum; TEER: trans-epithelial electrical resistance; HBSS: Hank's balanced salt solution; HEPES: n-(2-hydroxyethyl) piperazine-n-(2-ethanosulfonic acid); PBS: phosphate buffered saline; *P*_app_: apparent permeability coefficient; ODS: octadecylsilane.

## Competing interests

The authors declare that they have no competing interests.

## Authors' contributions

PT and WG conceived the study design, synthesized the compounds 1, 2, 3 and D, performed HPLC and UV analysis, and drafted the manuscript. JK and PO designed and performed the Caco-2 cell experiment and helped analyze the data. All authors read and approved the final version of the manuscript.

## References

[B1] Amatayakul T, Cannon JR, Dampawan P, Dechatiwong T, Giles RG, Huntrakul C, Kusamran K, Mokkhasamit M, Raston CL, Reutrakul V, White AH (1979). Chemistry and crystal structures of some constituents of *Zingiber cassumuar*. Aust J Chem.

[B2] Panthong A, Kanjanapothi D, Niwatananun V, Tuntiwachwuttikul P, Reutrakul V (1990). Anti-inflammatory activity of compounds isolated from *Zingiber cassumunar*. Planta Med.

[B3] Kanjanapothi D, Soparat P, Panthong A, Tuntiwachwuttikul P, Reutrakul V (1987). A uterine relaxant compound from *Zingiber cassumunar*. Planta Med.

[B4] Nualkaew S, Gritsanapan W, Petereit F, Nahrstedt A (2004). New fatty acid esters originate during storage by the interaction of components in Prasaplai, a Thai traditional medicine. Planta Med.

[B5] Tangyuenyongwatana P, Gritsanapan W (2008). A study on artifacts formation in the Thai traditional medicine Prasaplai. Planta Med.

[B6] Poomchusri NT (1973). Ayurvedic Study.

[B7] Tangyuenyongwatana P, Gritsanapan W (2007). Biological evaluations of fatty acid esters originated during storage of Prasaplai, a Thai traditional medicine. Nat Prod Res.

[B8] Hostetler KY, Parker S, Sridhar CN, Martin MJ, Li JL, Stuhmiller LM, Vanwijk GM, van den Bosch H, Gardner MJ, Aldern KA, Richman DD (1993). Acyclovir diphosphate dimyristoylglycerol: a phospholipid prodrug with activity against acyclovir-resistant herpes simplex virus. Proc Natl Acad Sci.

[B9] Yamashita S, Furubayashi T, Kataoka M, Sakane T, Sezaki H, Tokuda H (2000). Optimized conditions for prediction of intestinal drug permeability using Caco-2 cells. Eur J Pharm Sci.

[B10] Artursson P, Karlsson J (1991). Correlation between oral drug absorption in humans and apparent drug permeability coefficients in human intestinal epithelial (Caco-2) cells. Biochem Biophys Res Commun.

[B11] Lambert WJ (1993). Modeling oil-water partitioning and membrane permeation. using reversed-phase chromatography. J Chromatogr A.

[B12] Dorsey J, Khaledi M (1993). Hydrophobicity estimation by reversed-phase liquid chromatography. J Chromatogr A.

